# Lung Transplantation with Planned Delayed Chest Closure in Severe Pectus Excavatum after Multiple Pleurodeses: A Case Report

**DOI:** 10.70352/scrj.cr.26-0685

**Published:** 2026-10-31

**Authors:** Yusuke Nakano, Tatsuaki Watanabe, Hirotsugu Notsuda, Takuya Ikushima, Sakiko Kumata, Ken Onodera, Yui Watanabe, Takashi Hirama, Takaya Suzuki, Hisashi Oishi, Takashi Kochi, Yoshinori Okada

**Affiliations:** 1 Department of Thoracic Surgery, Institute of Development, Aging and Cancer, Tohoku University, Sendai, Miyagi, Japan; 2 Division of Organ Transplantation, Tohoku University Hospital, Sendai, Miyagi, Japan; 3 Department of Plastic and Reconstructive Surgery, Sendai City Hospital, Sendai, Miyagi, Japan

**Keywords:** pectus excavatum, pectus bar, lung transplantation, delayed chest closure, pleurodesis

## Abstract

**INTRODUCTION:**

Lung transplantation combined with pectus excavatum repair has been reported in selected patients with severe pectus excavatum to expand the restricted thoracic cavity and facilitate graft accommodation. However, the safe implementation of this surgical strategy may be challenging in patients with extensive pleural adhesions after repeated pleurodeses, because of the increased risk of postoperative bleeding and re-thoracotomy.

**CASE PRESENTATION:**

A 15-year-old boy with interstitial lung disease, secondary pulmonary hypertension, severe pectus excavatum, and multiple prior bilateral pleurodeses underwent deceased-donor bilateral lung transplantation using a planned delayed chest closure (DCC) strategy. Preoperative CT showed marked thoracic deformity with a Haller index of 9.1 and a leftward mediastinal shift. Because extensive pleural adhesions were expected to complicate graft implantation and hemostasis and increase the risk of re-thoracotomy for postoperative bleeding, a 2-stage strategy consisting of planned DCC followed by pectus bar placement at definitive chest closure was established at the time of transplant listing in collaboration with an external pectus specialist. Bilateral lung transplantation was performed under venoarterial extracorporeal membrane oxygenation support, followed by temporary chest closure. At the second-stage operation on POD 2, 2 pectus bars were placed, and additional graft volume reduction was performed, allowing definitive chest closure without hemodynamic compromise. The patient was discharged home on POD 69 without wound complications or implant-related infections.

**CONCLUSIONS:**

This case suggests that lung transplantation may be feasible in carefully selected patients with severe pectus excavatum and extensive pleural adhesions, with appropriate multidisciplinary planning. A preplanned 2-stage strategy incorporating DCC and pectus bar placement at definitive chest closure represents a potential option for such complex cases.

## Abbreviations

DCC
: delayed chest closure
FEV_₁_
: forced expiratory volume in 1 second
FVC
: forced vital capacity
PGD
: primary graft dysfunction
V-A ECMO
: venoarterial extracorporeal membrane oxygenation
mMRC
: modified Medical Research Council

## INTRODUCTION

Severe chest wall deformity has been considered a risk factor in lung transplantation because restricted intrathoracic space may compromise graft expansion and function.^1)^ In cases with severe pectus excavatum, however, pectus bar placement with or without a modified Ravitch repair at the time of chest closure after lung transplantation has been increasingly reported as a means of expanding the restricted thoracic cavity and preserving optimal graft function.^2–8)^ Despite this favorable trend toward expanding the indications for lung transplantation in patients with severe chest wall deformity, the published evidence remains limited to isolated case reports and small case series.

Dense intrathoracic adhesions after repeated pleurodeses are recognized as another risk factor in lung transplantation,^1)^ as they may increase intraoperative bleeding, prolong operative time, and increase the risk of re-exploration for hemostasis. In patients with severe pectus excavatum who have undergone repeated pleurodeses, careful consideration is therefore required regarding not only candidacy for lung transplantation but also the timing of pectus bar placement, because such patients may have an increased risk of re-thoracotomy.

Here, we report a case of successful bilateral lung transplantation in a 15-year-old boy with interstitial lung disease as a sequela of cytomegalovirus pneumonia in infancy, severe pectus excavatum, and a history of multiple bilateral pleurodeses for recurrent pneumothorax. We performed planned DCC, followed by pectus bar placement during definitive chest closure on POD 2. In addition, we reviewed the 8 previously reported cases of lung transplantation combined with pectus bar placement to provide an overview of the outcomes of this surgical strategy.

## CASE PRESENTATION

At the time of transplantation, the patient was a 15-year-old boy (height, 153 cm; weight, 27.2 kg) with interstitial lung disease secondary to cytomegalovirus pneumonia in infancy. He was born at 37 weeks and 5 days of gestation by spontaneous vaginal delivery, with a birth weight of 3034 g and Apgar scores of 9 and 10 at 1 and 5 minutes, respectively. At 3 months of age, he developed cytomegalovirus pneumonia, which resulted in bilateral emphysematous and fibrotic lung changes requiring home oxygen therapy. Whether the infection was congenital or postnatally acquired, as well as the details of immunodeficiency testing, could not be confirmed. His subsequent course, including the period after transplantation, showed no overt susceptibility to infection. He subsequently developed recurrent pneumothoraces between 2 and 13 years of age, with 5 episodes on the right side requiring 3 pleurodeses, and 4 episodes on the left side requiring 1 pleurodesis. At 11 years of age, secondary pulmonary hypertension was diagnosed, and tadalafil was initiated at 16 mg/day. Despite treatment, his respiratory status progressively deteriorated, and he was referred to our institution at 14 years of age for lung transplantation evaluation.

On physical examination, severe pectus excavatum with marked anterior chest wall depression was evident (**Fig. 1A**). Chest CT demonstrated extensive bilateral emphysematous and fibrotic changes consistent with the underlying lung disease (**Fig. 2A**). In addition, pectus excavatum with marked chest wall deformity and a leftward mediastinal shift was observed (Haller index, 9.1) (**Figs. 2C** and **3A**). At the time of listing, his mMRC dyspnea grade was 2, and his 6-minute walk distance was 279 m, supporting his ability to participate in rehabilitation. At transplant listing (age, 14 years; height, 142.2 cm; weight, 25 kg), pulmonary function testing showed a FVC of 0.92 L (34.0% predicted) and a FEV_1_ of 0.86 L (35.3% predicted). Percent-predicted values were calculated using the patient’s age and height at each assessment. CT volumetry using the SYNAPSE VINCENT system (Fujifilm Corporation, Tokyo, Japan) demonstrated a total lung volume of 1.54 L. Arterial blood gas analysis on 3 L/min oxygen via nasal cannula showed a PaO_2_ of 78.0 mmHg and a PaCO_2_ of 54.6 mmHg. Right heart catheterization demonstrated secondary pulmonary hypertension, with a mean pulmonary arterial pressure of 32 mmHg.

**Fig. 1:**
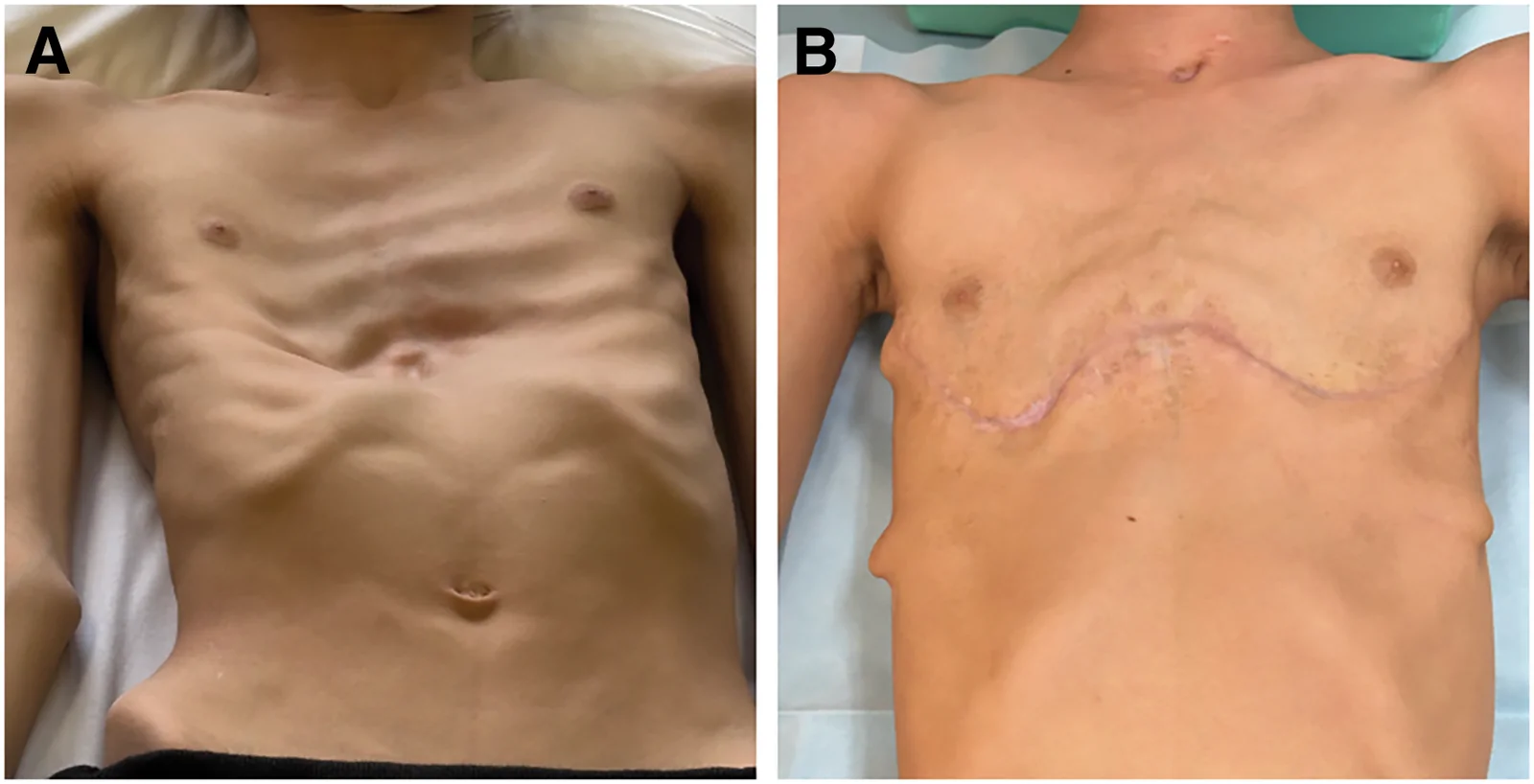
Preoperative and 10-month postoperative clinical photographs. (**A**) Preoperative appearance showing severe pectus excavatum. (**B**) Appearance 10 months after bilateral lung transplantation, pectus bar placement, and definitive chest closure.

**Fig. 2:**
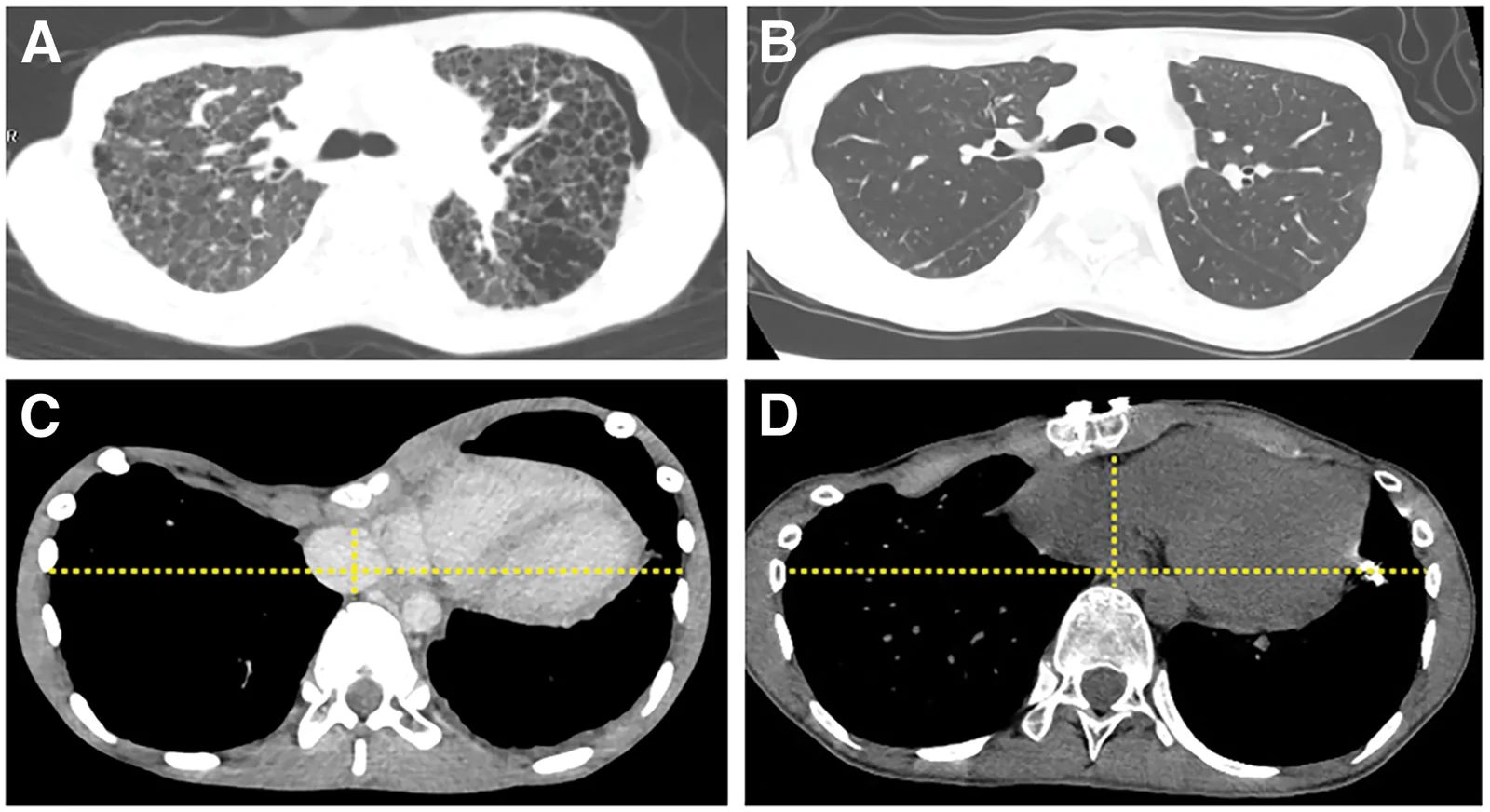
Chest CT before transplantation and at 10 months after transplantation. (**A**) Preoperative lung-window image showing emphysematous and fibrotic changes with a small left pneumothorax. (**B**) Postoperative lung-window image showing no abnormal findings in the lung grafts. (**C**) Preoperative axial CT image showing severe pectus excavatum (Haller index, 9.1) and a leftward mediastinal shift. (**D**) Postoperative axial CT image showing improved chest wall deformity and mediastinal position (Haller index, 4.9). Dotted lines in panels **C** and **D** indicate the diameters used to calculate the Haller index.

**Fig. 3:**
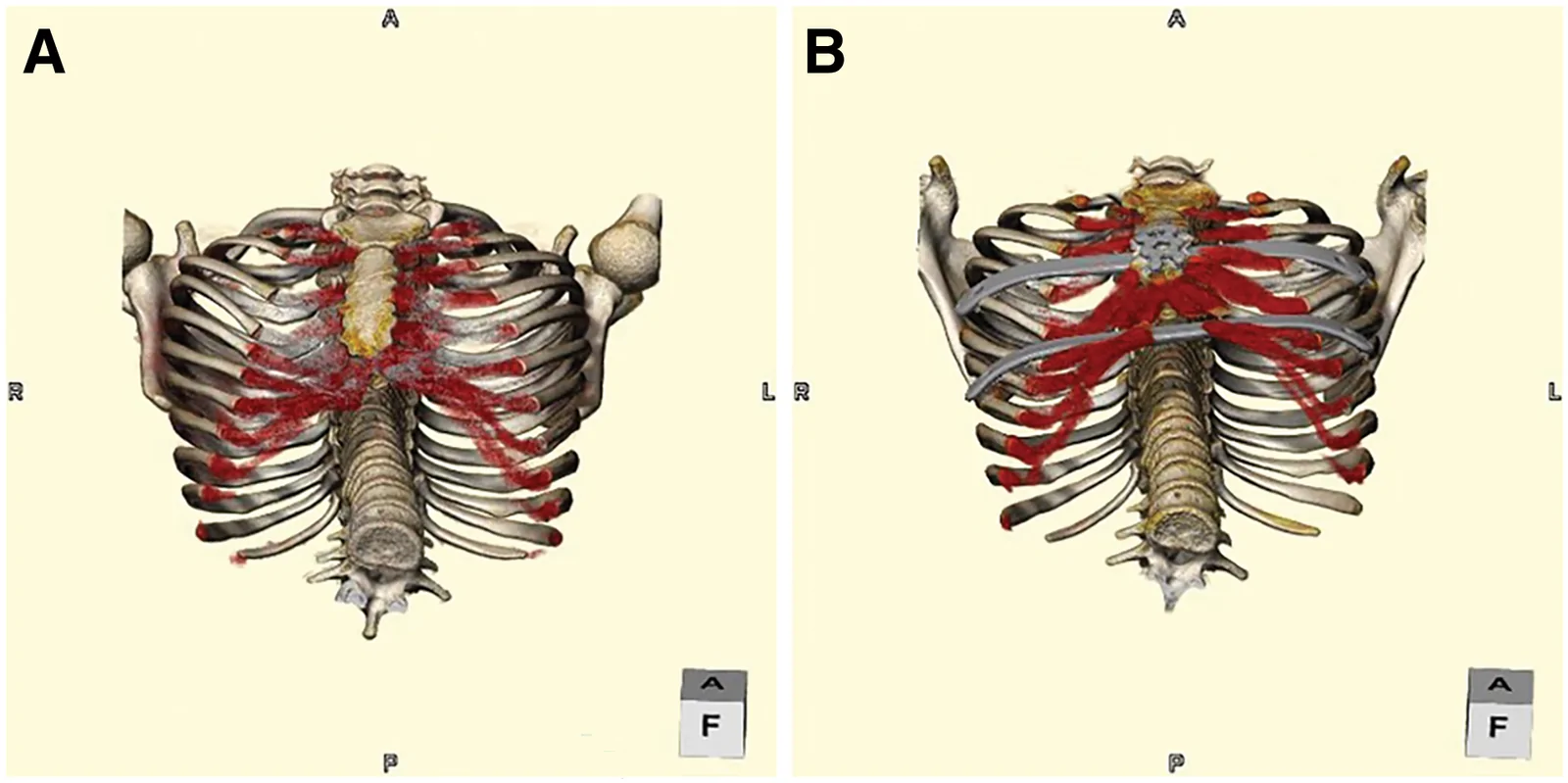
3D CT reconstructions before and after pectus bar placement. (**A**) Preoperative reconstruction showing marked anterior chest wall depression. (**B**) Postoperative reconstruction showing correction of the deformity and an increased anteroposterior thoracic dimension. Reddish-brown areas indicate the costal cartilages.

Because this patient had 2 major risk factors for lung transplantation—severe chest wall deformity and multiple prior bilateral pleurodeses, the latter potentially increasing the risk of re-thoracotomy for hemostasis—we carefully discussed both his candidacy for lung transplantation and the operative strategy. During a multidisciplinary conference that included an external plastic surgeon specializing in pectus excavatum repair, we decided to perform planned DCC, with pectus bar placement during definitive chest closure.

After 343 days on the waiting list, a suitable deceased-donor lung allograft became available. Before transplantation, his mMRC grade had increased to 4. Nevertheless, at the time of the donor offer, the transplant team considered that potential for postoperative recovery remained, taking into account his motivation and family support. The donor predicted FVC-to-recipient predicted FVC ratio was 116%, suggesting moderate graft oversizing. Given this ratio and the markedly restricted thoracic cavity, graft size reduction was anticipated at the time of donor acceptance. Through a clamshell thoracotomy, dense pleural adhesions were encountered in the right hemithorax, where 3 pleurodesis procedures had previously been performed, whereas moderate adhesions were observed in the left hemithorax, where 1 pleurodesis procedure had been performed. Following adhesiolysis, central V-A ECMO was established, and sequential bilateral lung transplantation was performed using standard techniques, first on the right (ischemic time, 408 minutes) and then on the left (ischemic time, 482 minutes). After successful weaning from V-A ECMO 50 minutes after reperfusion of the second lung, the chest was temporarily closed with an Esmarch bandage as part of the planned DCC strategy (**Fig. 4A**). The operative time was 9 hours 47 minutes, and the estimated blood loss was 1001 mL. Standard immunosuppression and routine infection prophylaxis with piperacillin/tazobactam, micafungin, and ganciclovir were administered according to our institutional protocol. In addition, daptomycin was administered until completion of definitive chest closure.

**Fig. 4:**
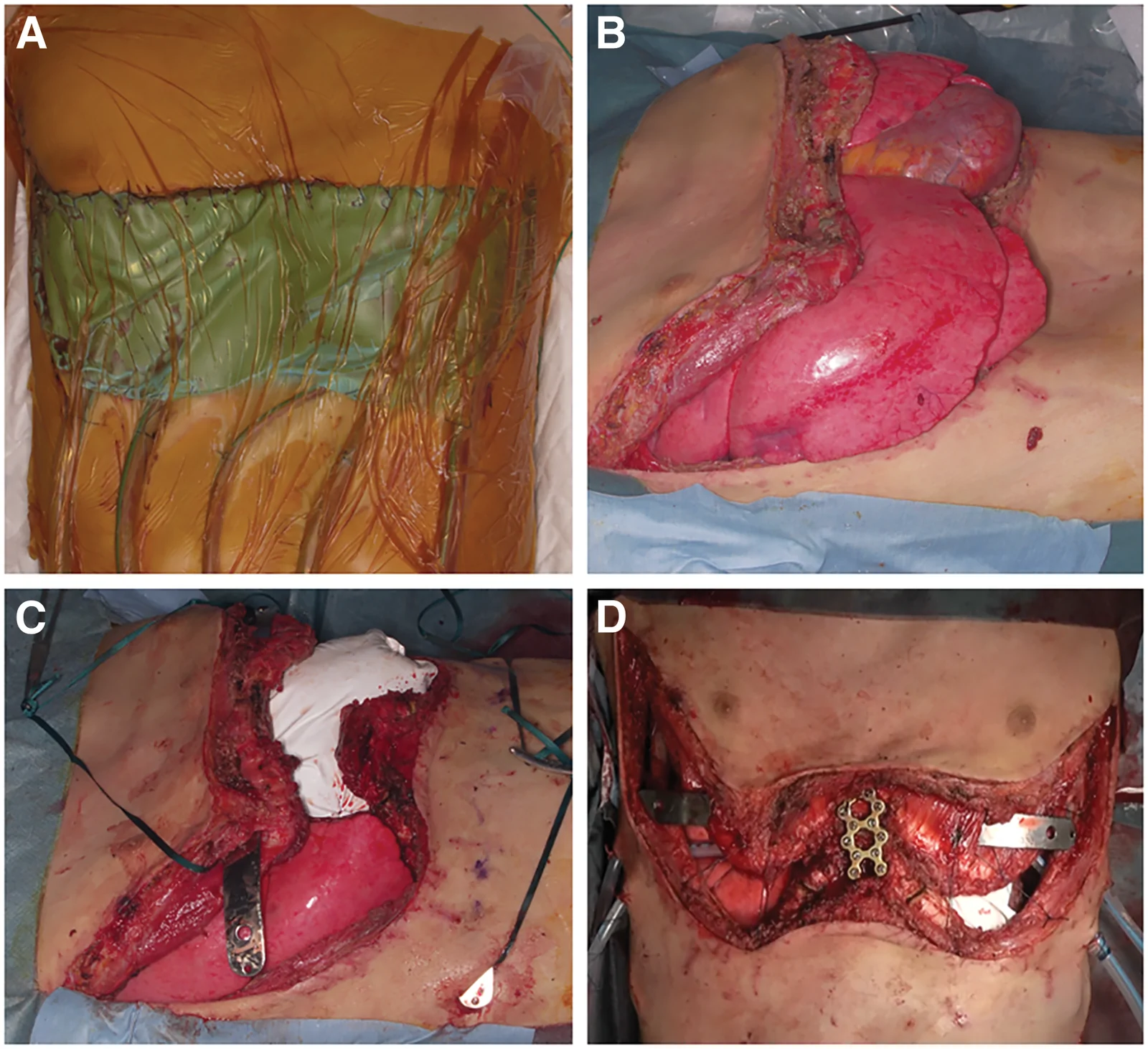
Intraoperative findings. (**A**) Temporary chest closure with an Esmarch bandage after the initial transplantation. (**B**) Relatively oversized grafts in the restricted thoracic cavity at the second-stage operation. (**C**) Placement of 2 pectus bars and a pericardial sheet. (**D**) Definitive chest closure reinforced with a sternal plate.

On POD 2, hemostasis was satisfactory, the PaO_2_/FiO_2_ ratio was 322, and CT showed minimal dependent atelectasis without marked pulmonary edema or evident anastomotic abnormalities. After a multidisciplinary discussion, pectus excavatum repair and definitive chest closure were undertaken jointly with the external pectus specialist, considering these findings and the specialist’s availability. The decision also reflected concerns that prolonged temporary chest closure would require continued deep sedation, thereby delaying rehabilitation, and could increase the risk of intrathoracic infection. At the time of the second-stage operation, the grafts still appeared relatively oversized for the limited thoracic cavity (**Fig. 4B**). Two 23-cm pectus bars (CHEST WAY; SOLVE Co., Ltd., Yokohama, Japan) were placed at the third and fifth intercostal spaces (**Fig. 4C**). The extent of graft size reduction was determined after bar placement. To preserve lung parenchyma, wedge resections of the lingula and right middle lobe were performed sequentially. Hemodynamic instability during trial chest approximation prompted anatomical resection of the remaining right middle lobe. Persistent instability on repeat approximation then prompted an additional wedge resection of the peripheral portion of segment 8 of the left lower lobe, which was directly compressing the heart, allowing definitive chest closure with stable hemodynamics.

To reduce the risk of cardiac injury at the time of subsequent pectus bar removal, a 0.1-mm-thick pericardial sheet (GORE PRECLUDE Pericardial Membrane; Nihon Gore LLC, Tokyo, Japan) was sutured over the pericardium (**Fig. 4C**). The sternum was then fixed with a sternal plate (Stryker, Kalamazoo, MI, USA) (**Fig. 4D**). The operative time was 3 hours 26 minutes, and the blood loss was minimal.

After definitive closure on POD 2, an epidural catheter was placed following confirmation of an adequate platelet count and coagulation status. Analgesia comprised epidural levobupivacaine, a continuous morphine infusion, and enteral acetaminophen. Opioids were stopped on day 7 and the catheter was removed on day 8, with only as-needed acetaminophen thereafter. Neither nonsteroidal anti-inflammatory drugs nor gabapentinoids were used. Management followed our usual analgesic approach after lung transplantation. No additional analgesic measures were required specifically because of the pectus bars, and pain did not limit rehabilitation.

PGD grades at 24, 48, and 72 hours after reperfusion of the second lung were 1, 1, and 2, respectively; the 72-hour assessment was performed after definitive chest closure. A tracheostomy was performed on POD 8. He was weaned from mechanical ventilation on POD 21 and discharged home on POD 69. No wound complications or implant-related infections occurred. At 10 months after transplantation (age, 16 years; height, 154.0 cm; weight, 29.1 kg), pulmonary function testing showed an FVC of 1.27 L (36.9% predicted) and an FEV_1_ of 1.21 L (38.9% predicted). His chest appearance improved significantly (**Fig. 1B**). CT volumetry demonstrated a total lung volume of 2.2 L. The Haller index improved to 4.9 (**Figs. 2D** and **3B**), and no abnormal findings were observed in the lung grafts (**Fig. 2B**). Arterial blood gas analysis on room air showed a PaO_2_ of 101 mmHg and a PaCO_2_ of 37.7 mmHg. Echocardiography at transplant listing and at 6 and 10 months after transplantation showed left ventricular ejection fractions of 66%, 70%, and 55%, respectively, and right ventricular fractional area changes of 32.8%, 34.0%, and 22.2%, respectively. These findings did not demonstrate consistent improvement in cardiac function, although serial comparison was limited by suboptimal image quality. At the 1-year follow-up, his clinical condition remained stable. No clinical episodes of acute cellular rejection or antibody-mediated rejection occurred during the first postoperative year. No clinically significant infections were observed during this period. He returned to school, and a pectus bar removal is planned for 3 years after transplantation.

## DISCUSSION

Commonly considered indications for the Nuss procedure include morphological severity, a Haller index greater than 3.25, clinical symptoms, cardiopulmonary impairment, and psychosocial burden.^9)^ In lung transplantation, however, the need for chest wall reconstruction should be guided less by the Haller index itself than by whether the deformity, mediastinal deviation, and restricted thoracic space may compromise graft accommodation or prevent safe definitive chest closure.^2)^ This concept is consistent with the expert consensus document on donor–recipient size matching, which emphasizes the importance of recipient chest anatomy and recommends considering pectus excavatum repair at the time of transplantation in patients with severe pectus excavatum.^10)^

The main technical challenges in the present case were severe pectus excavatum and anticipated dense pleural adhesions after multiple bilateral pleurodeses. Therefore, planned DCC was incorporated into the operative strategy from the outset, with pectus bar placement deferred until postoperative hemostatic stability had been confirmed. This strategy also allowed sufficient time for preparation of the pectus bars and facilitated the planned participation of an external plastic surgeon experienced in pectus excavatum repair.

According to our literature review, 8 previously reported patients underwent lung transplantation combined with pectus excavatum repair; together with the present case, 9 patients have been described (**Table 1**).^2–8)^ Five of the nine patients were pediatric. The indications for lung transplantation were interstitial lung disease, including idiopathic pulmonary fibrosis, in 6 patients (IPF, n = 3; other ILD, n = 3), pulmonary alveolar proteinosis in 2, and bronchiolitis obliterans syndrome after hematopoietic stem cell transplantation in one. Eight patients underwent deceased-donor transplantation, whereas one underwent living-donor segmental lung transplantation.^5)^ One or two pectus bars were used in all 9 patients, with an additional modified Ravitch procedure performed in 1 patient with marked asymmetric chest wall deformity.^3)^ The preoperative Haller index ranged from 3.32 to 9.5 and improved in all patients for whom postoperative values were available. All patients were alive at the latest reported follow-up, and no persistent functional limitations were reported, although the duration of follow-up varied considerably.

**Table 1:** Reported cases of lung transplantation combined with pectus excavatum repair

Author, year	Age/sex	Disease	LTx procedure	Prior thoracic procedure/pleurodesis	Chest closure	Pectus repair	Haller index, pre/post	Outcome
Valdivia et al., 2019^6)^	38/M	IPF	Bilateral	Yes, prior unilateral surgery	Primary	One pectus bar	4.3 → 2.4	Alive, 210 days
Zhang et al., 2020^7)^	39/F	BOS	Bilateral	NR	Primary	One pectus bar*	3.32 → NR	Alive, 4 years
Matilla et al.**, 2021^3)^	9/F	PAP	Bilateral with graft size reduction	NR	Primary	Modified Ravitch + 2 pectus bars	9.5 → 3.5	Alive, 1 year
Rahimi et al., 2021^4)^	38/F	IPF	Bilateral with graft size reduction	NR	Primary	One pectus bar	5.8 → 2.2	Alive, 6 years
Rahimi et al., 2021^4)^	14/M	PAP	Bilobar	NR	Primary	One short pectus bar	5.5 → 2.7	Alive, 5 years
Tanaka et al., 2023^5)^	10/M	ILD	Living-donor segmental	NR	Delayed, unplanned	Two pectus bars	8.8 → NR	Alive, 1 year
Alzahrani et al., 2025^2)^	36/F	IPF	Bilateral	Yes, prior bilateral pleurodesis	Delayed, planned	Two pectus bars*	6.7 → 2.9	Alive, 910 days
Schwab et al., 2025^8)^	5/F	ILD	Bilateral	NR	Primary	Two pectus bars + partial chondrotomies	5.9 → NR	Alive, 8 months
Present case	15/M	ILD	Bilateral with graft size reduction	Yes, prior bilateral pleurodesis	Delayed, planned	Two pectus bars	9.1 → 4.9	Alive, 1 year

*Pectus bar removal was reported 2 years after transplantation by Zhang et al. and on POD 659 by Alzahrani et al.

**The case reported by Matilla et al. corresponds to the third case in the series described by Rahimi et al.

No persistent functional limitations were described in the available follow-up reports.

BOS, bronchiolitis obliterans syndrome; F, female; ILD, interstitial lung disease; IPF, idiopathic pulmonary fibrosis; LTx, lung transplantation; M, male; NR, not reported; PAP, pulmonary alveolar proteinosis; post, postoperative; pre, preoperative

Among the 9 reported cases, primary chest closure was achieved in 6 patients, whereas DCC was required in 3. Tanaka et al. placed the pectus bars during the initial living-donor lung transplantation; however, this patient required unplanned DCC.^5)^ Alzahrani et al. described a closely comparable case of severe pectus excavatum with repeated bilateral pleurodeses that was managed with planned DCC and staged Nuss bar placement.^2)^ Our case adds to the experience of managing a markedly underweight pediatric recipient with a Haller index of 9.1 who required additional graft volume reduction after chest wall correction. Further delay might have reduced graft edema and the extent of lung resection, although the amount of lung parenchyma that could have been preserved remains uncertain.

Although no adverse events directly related to pectus bar placement were reported among the 9 patients, potential complications associated with the Nuss procedure should be carefully considered in lung transplant recipients. Adequate postoperative analgesia is essential for coughing, sputum clearance, early mobilization, and pulmonary rehabilitation.^3,8)^ Bar-related complications, including bar displacement, surgical site infection, and bar exposure, may be particularly consequential in lung transplant recipients receiving immunosuppressive therapy.^9)^ Furthermore, pectus bar removal was documented in only 2 patients (Zhang et al. and Alzahrani et al.),^2,7)^ and the optimal timing and long-term safety of bar removal in immunosuppressed recipients remain uncertain. Therefore, careful pain control, infection prevention, secure bar fixation, appropriate timing of bar removal, and close follow-up are warranted.

## CONCLUSIONS

The present case highlights the importance of chest-closure-oriented planning, including a planned DCC strategy, in lung transplantation for patients with severe chest wall deformity and a hostile pleural space due to extensive pleural adhesions.

## DECLARATIONS

### Availability of data and materials

The data supporting the conclusions of this article are included within the article.
